# Associations Between Obesity and the Severity of Occupational Allergic Rhinitis: A Cross-Sectional Study

**DOI:** 10.3390/ijerph22101531

**Published:** 2025-10-06

**Authors:** Imène Kacem, Amen Moussa, Chaima Sridi, Amene Fki, Mohamed Ajmi, Maissa Thabet, Olfa El Maalel, Maher Maoua, Mohamed Kahloul, Najib Mrizek

**Affiliations:** 1 Faculty of Medicine of Sousse, University of Sousse, Sousse 4000, Tunisia; 2 Occupational Medicine Department, Farhat Hached University Hospital, Sousse 4000, Tunisia; 3 Laboratoire de Recherche (LR19SP03: Etude des Risques et Perspectives de Prévention des Maladies non Transmissibles en Milieu Professionnel), Sousse 4000, Tunisia; 4 Occupational Medicine Department, Faculty of Medicine of Monastir, Monastir 5019, Tunisia; 5 Occupational Medicine Department, Sahloul University Hospital, Sousse 4000, Tunisia; 6 Anesthesia and Intensive Care Department, Sahloul University Hospital, Sousse 4000, Tunisia; 7 Internal Medicine Department, Farhat Hached University Hospital, Sousse 4000, Tunisia

**Keywords:** occupational allergic rhinitis, obesity, severity, risk factors

## Abstract

Introduction: Occupational allergic rhinitis (OAR) is a common respiratory condition that can lead to varying degrees of symptom severity, significantly impacting workers’ quality of life and productivity. While occupational risk factors are well established, the influence of nonoccupational factors, such as obesity, that contribute to OAR severity remains largely unexplored. Aims: This study aims to study the association between obesity and the severity of OAR. Methods: A cross-sectional analytical study was conducted among patients diagnosed with OAR at the Occupational Medicine Department of Farhat Hached University Hospital of Sousse. It combines a retrospective review of medical records (2013–2021) with prospective structured telephone interviews (January–March 2023). Data were collected from medical records and supplemented with telephone interviews. The severity of OAR was assessed via the PAREO score and rhinomanometry results. Results: A total of 196 patients were included. The mean age was 39.69 ± 7.92 years, with a sex ratio of 0.53. The most frequently reported symptoms were nasal obstruction (78.6%) and sneezing (88.8%). The mean PAREO score was 5.78 ± 1.61, with severe OAR reported in 59.2% of the patients. Obesity was significantly associated with increased severity of OAR symptoms (*p* < 0.001; OR = 5.4; 95% CI [2.6–11.1]), a finding confirmed after adjustment for variables such as age, sex, and occupational seniority. Conclusion: Obesity appears to be a modifiable risk factor influencing OAR severity. Integrating weight management strategies into the treatment of OAR patients may contribute to significant symptom relief and improved quality of life. Further longitudinal studies are needed to confirm these findings and explore the underlying mechanisms involved.

## 1. Introduction

Occupational rhinitis (OR) is an inflammatory disease of the nose. It is characterized by intermittent or persistent symptoms (e.g., nasal congestion, sneezing, rhinorrhea, and itching) and/or variable nasal airflow limitation and/or hypersecretion. Its underlying causes are attributable to the particular work environment and not to stimuli encountered outside the workplace. OR can be either allergic or nonallergic. Nonallergic ORs are triggered by irritants and nonimmunological reactions, whereas occupational allergic rhinitis (OAR) is caused by immunological sensitization to workplace allergens [1,2,3].

Allergic rhinitis affects 10 to 30% of the population in developed countries, and its prevalence is increasing in developing countries [4]. A substantial body of research has revealed that 15% of the working population is affected by OR, which constitutes 4% of occupational respiratory diseases [5]. Despite its higher prevalence than occupational asthma, OAR remains underdiagnosed and undertreated. Patients with mild-to-moderate symptoms are less likely to seek medical advice [6,7]. Consequently, OAR is associated with a negative impact on quality of life (QOL) and work ability [5], resulting in functional limitations, reduced productivity and substantial financial hardship, mainly in its severe clinical presentation [8,9,10,11,12].

While genetic susceptibility and family history of allergies are recognized as major risk factors for OAR, other factors should be considered, such as exposure to allergens, indoor and outdoor pollution, and dietary factors [4]. Therefore, further research is needed to identify OAR severity risk factors, which are mainly modifiable, such as obesity. This major global public health concern, whose prevalence has tripled since 1975 [13], is multifactorial. It is associated with several respiratory disorders, including asthma, obstructive sleep apnea, and chronic rhinosinusitis, largely due to systemic inflammation, mechanical airway obstruction, and altered immune responses [14].

While the impact of obesity on respiratory diseases has been extensively assessed, its specific role in exacerbating occupational allergic rhinitis (OAR) symptoms remains under investigation. The systemic inflammation and immune dysregulation linked to obesity suggest a potential mechanism for amplifying OAR. Specifically, obesity-induced alterations in airway physiology and chronic low-grade inflammation may contribute to increased nasal hyperreactivity and heightened allergic responses in occupational settings [15]. The interest in allergic rhinitis in the workplace is justified by its frequency, particularly due to occupational exposure, its repercussions for employees, and its impact on productivity. Identifying factors that can aggravate its progression, such as obesity, which is increasingly common, could lead to the identification of preventive targets. The present study therefore aimed to analyze the relationship between obesity and the severity of OAR in a cohort of Tunisian workers.

## 2. Materials and Methods

### 2.1. Study Design

This analytical cross-sectional study was conducted in 2023 at the Occupational Medicine Department of Farhat Hached University Hospital, Sousse. It combines a retrospective review of medical records (2013–2021) with prospective structured telephone interviews (January–March 2023).

### 2.2. Participants and Sampling

This study enrolled patients who were diagnosed with OAR between January 2013 and December 2021, who were active employees during data collection and who had undergone anterior rhinomanometry. OAR was diagnosed via a combination of clinical evaluation and occupational history. Specifically, participants were classified as having OAR if they met typical nasal symptoms (sneezing, rhinorrhea, congestion, and itching) occurring during work hours and improving on their days off or vacation. Individuals were excluded from the study if their medical records were incomplete, if they were unreachable despite three attempts to contact them via telephone, or if they withdrew their informed consent. Participants with a prior diagnosis of nasal polyposis, obstructive sleep apnea syndrome (OSAS), or chronic rhinosinusitis with nasal polyps were excluded from the study.

### 2.3. Data Collection

At the time of occupational health consultations, informed consent for research was not routinely sought, as this study was not planned during routine clinical practice. Subsequently, structured telephone interviews (January–March 2023) were conducted to obtain oral informed consent from all participants and supplement incomplete clinical data. The real-time data collected during the occupational visit included anthropometric measurements, rhinoscopy, rhinomanometry, and initial PAREO scores. The collected variables included sociodemographic characteristics (e.g., age, sex, educational qualifications, marital status), lifestyle habits (e.g., smoking, alcohol consumption, physical activity), occupational characteristics (e.g., sector of activity, occupation, occupational seniority, use of protective respiratory equipment), and medical data (e.g., personal and family allergy history, body mass index (BMI), OAR symptoms, clinical examination findings, rhinomanometry results). Anterior rhinoscopy was performed during the ENT examination via a nasal speculum and direct illumination; nasal endoscopy was not systematically performed. The severity of OAR was assessed through the P.A.R.E.O. score and rhinomanometry. P.A.R.E.O. stands for Pruritus, Anosmia, Rhinorrhea, Eternuation [sternutation], and Obstruction. It is a validated scale evaluating the severity of functional signs associated with OAR, with each symptom scored from 0 to 2 (0 = absent, 1 = mild or bothersome, 2 = present and severe), yielding a total score from 0 to 10. Rhinomanometry was performed on all patients to provide objective measurements.

### 2.4. Operational Variables

Severe OAR was defined as a PAREO score exceeding 5 or a rhinomanometry value less than 500 mL/s. Moderate OAR was defined as RNT 500–700 mL/s, mild OAR was defined as RNT between 700 and 870 mL/s, and normal nasal function was defined as RNT greater than 870 mL/s. The classification of obesity is based on the World Health Organization (WHO) criteria. Overweight corresponds to a BMI ranging between 25 and 29.9 kg/m^2^, and obesity is defined as a BMI ≥ 30 kg/m^2^.

### 2.5. Data Analysis

The data were analyzed via SPSS version 25.0. Qualitative variables are reported as numbers and percentages. Quantitative variables are reported as the means ± standard deviations. The chi-square test and Fisher’s exact test were applied to compare categorical variables, whereas Student’s *t* test or the Mann–Whitney U test was used for continuous variables. Multivariate logistic regression was performed to estimate adjusted odds ratios (aORs) with 95% confidence intervals (CIs), controlling for potential confounders. The dependent factor was OAR severity. Spearman’s rank correlation coefficient (ρ) was computed to assess the association between the subjective PAREO symptom score and objective rhinomanometric values. Statistical significance was set at *p* ≤ 0.05.

### 2.6. Ethical Considerations

This study was conducted in accordance with the ethical principles of the Declaration of Helsinki. Participation was voluntary, and informed consent was obtained from all participants during the initial telephone contact (January–March 2023) prior to conducting the interviews.

Anonymity and confidentiality were ensured throughout the data collection and analysis process. The study was approved by the institutional ethics committee of the Sahloul University Hospital.

## 3. Results

**Figure 1:**
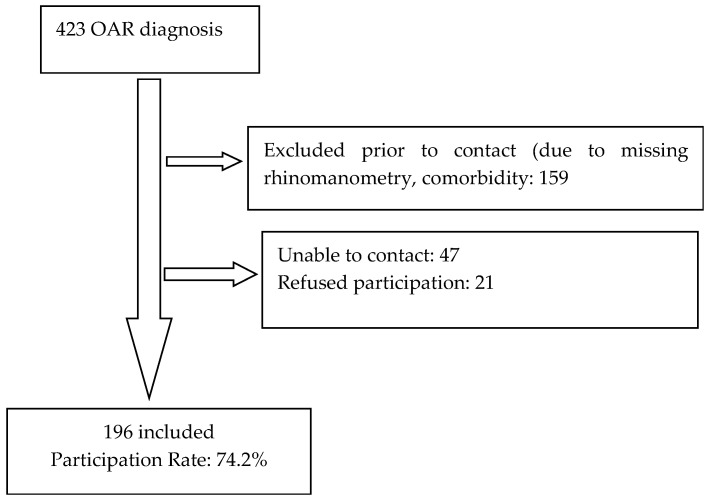
Flow chart.

A total of 196 patients were included in this study from the 264 eligible patients (see Figure 1), representing a participation rate of 74.2%.

The mean age of the participants was 39.7 ± 7.9 years. The sex ratio was 0.53 (65.3% were female). The participants were predominantly married (75%) and had completed primary education in 69.4% of the cases. Among the participants, 19.4% were active smokers, and 27% reported engaging in regular physical activity. The most common occupational sectors were textile manufacturing (49.5%), electronics (14.8%), and food production (10.2%). The most prevalent occupational categories were blue-collar workers (69.4%), welders (5.6%) and technicians (3.6%). The average occupational seniority was 15.3 ± 8.1 years, and only 14.8% used respiratory protective equipment (Table 1).

Among the participants, 14.3% (n = 28) reported a familial history of allergic disease dominated by asthma (n = 17). A personal history of allergic disease was reported by 12.8% of the participants. The primary reported condition was asthma (n = 6). The mean body mass index (BMI) was 28.07 ± 4.95 kg/m^2^, and 35.2% of the participants were classified as obese (Table 2).

The most frequently reported OAR symptoms were nasal obstruction (78.6%), sneezing (88.8%), rhinorrhea (67.8%), and pruritus (85.2%) (Table 3).

The mean PAREO score was 5.8 ± 1.6, and severe OAR was reported in 59.2% of the patients. Rhinomanometry revealed normal findings in 43.4% of the patients. Severe nasal obstruction was reported in 27% of the patients (Table 4). However, a weak but significant inverse correlation was observed between the PAREO score and rhinomanometric values (Spearman’s ρ = −0.24, *p* = 0.001).

Table 5 shows the prevalence of different degrees of OAR in the different BMI groups.

A significant association was observed between obesity and the clinical severity of OAR (*p* < 0.001; OR = 5.4; 95% CI [2.6–11.1]). However, no significant association was found between obesity and severe nasal obstruction, as measured by rhinomanometry (*p* = 0.82).

Other significant risk factors included female sex (*p* = 0.027; OR = 1.9; 95% CI [1.07–3.5]), marital status (*p* = 0.003; OR = 2.7; 95% CI [1.4–5.2]), lack of physical activity (*p* = 0.006; OR = 2.4; 95% CI [1.2–4.6]), occupational seniority (*p* = 0.024), and delay in symptom onset (*p* = 0.036) (Table 6).

After multivariate analysis, obesity remained the strongest independent predictor of severe OAR (*p* < 0.001; aOR = 5.47; 95% CI [2.6–11.1]) (Table 7).

## 4. Discussion

This study suggests that obesity may exacerbate OAR symptoms, providing valuable insights into this under-researched area. In fact, few studies have assessed the association between obesity and OAR in the general population, and fewer have investigated this relationship in an occupational setting.

Our findings align with available research conducted in general populations and report a link between obesity and allergic rhinitis (AR) and/or a link between higher BMI and a greater prevalence and severity of AR [16,17,18,19,20]. These studies highlight the potential of obesity to modulate immune responses and promote inflammation, which are key factors in the development of AR.

However, other studies reported no association between BMI and AR [21,22]. These discrepancies may result from variations in study methodologies, population characteristics, and the criteria used to define AR severity. Notably, these investigations focused primarily on nonoccupational AR, where the triggers and environmental context differ significantly.

Distinguishing between general AR and OAR is imperative, as our study focused exclusively on the occupational field. OAR is a respiratory condition precipitated by an IgE-mediated immune hypersensitivity reaction to a particular occupational allergen [1]. A latency period ranging from several months to several years is typically required to achieve sensitization to this agent. Re-exposure to the allergen serves as a trigger, prompting an inflammatory immune response that is frequently accompanied by the infiltration of eosinophils into the airways. However, notably, OAR is not invariably associated with a predominant IgE response. In certain instances, it can be facilitated by IgG antibodies or alternative adaptive immune mechanisms. The inflammation underlying AR, irrespective of its etiology (occupational or environmental allergens), is a multifaceted process. Its intensity can be increased by various factors, including systemic conditions such as obesity.

The lack of association between obesity and severe nasal obstruction as measured by rhinomanometry (*p* = 0.82), despite a strong association with subjective symptom severity (PAREO; *p* < 0.001; OR = 5.47), suggests that obesity exacerbates OAR primarily through systemic inflammatory and neurosensory pathways rather than via anatomical narrowing of the nasal airway. Obesity induces a state of chronic low-grade inflammation characterized by elevated adipokines (e.g., leptin) and proinflammatory cytokines (e.g., TNF-α and IL-6) [15], which promote airway hyperreactivity, increase mucosal permeability, and amplify IgE-mediated responses to occupational allergens. Concurrently, obesity is associated with systemic immune dysregulation and altered microbiome composition, including reduced diversity and increased colonization by *Staphylococcus aureus* in the nasal cavity [23], which may disrupt local immune homeostasis and lower the threshold for allergic inflammation. Emerging evidence further supports the concept of the “gut–airway axis,” wherein obesity-driven gut dysbiosis and systemic metabolic disturbances propagate distant inflammatory signals to the upper airway mucosa [24,25,26,27]. These interconnected mechanisms of systemic inflammation, immune deviation, and microbiome dysbiosis likely converge to intensify symptom perception and neurogenic signaling (e.g., via trigeminal nerve sensitization), thereby explaining the pronounced discordance between objective airflow measurements and patient-reported burden in obese individuals with OAR. Thus, while obesity does not appear to cause structural obstruction, it significantly amplifies the subjective experience of OAR through multifactorial, systemically mediated pathways.

Other factors, such as age and exposure to different allergens, may influence the severity of AR. Jabri H et al. [28] highlighted the impact of persistent allergen exposure, poor treatment adherence, humidity, and smoking on AR management. A recent Tunisian comparative study [29] involving patients with AR reported that persistent allergic exposure, poor treatment adherence, and uncontrolled asthma were associated with the development of severe forms of AR. Consistent and correctly fitted respiratory protection serves as a vital barrier against inhaled occupational allergens, directly reducing nasal exposure, dampening local inflammation, and lowering the risk of sensitization and symptom development in workers at risk for OAR. Its integration into comprehensive occupational health programs is essential for primary prevention. Therefore, further multivariate analyses, particularly in occupational settings, are needed to better understand the real impact of each of these factors.

Our study also revealed that female sex and lack of physical activity were independent risk factors for severe OAR. These findings align with the available literature and suggest that hormonal influences and immune system variations may underlie sex disparities due to hormonal modulation of immune responses in women [15,28,30,31]. The association between sedentary behavior and heightened allergic inflammation has also been previously described, suggesting that regular physical activity may be a protective factor [25,32].

Finally, it is important to acknowledge the limitations of our study before any definitive conclusions can be drawn. The cross-sectional design precludes causal inference and raises the possibility of reverse causation (severe OAR leading to reduced activity and subsequent obesity). Other limitations include lack of allergen quantification, reliance on self-reported behaviors, potential recall bias from telephone interviews and survivorship/availability bias. The single-center nature of the study may limit the generalizability of the findings. OAR severity was assessed by a validated score and objective rhinomanometry, whereas other factors, such as the specific types of allergens encountered in the workplace, should have been considered. Consequently, further research, particularly longitudinal studies with larger populations, is necessary to validate our findings and elucidate the interactions of different factors contributing to OAR severity. Additionally, the dominance of textile workers in our sample may introduce potential biases. such as the healthy worker effect and distinct occupational exposures in the textile sector from those in other manufacturing or industrial sectors.

Owing to the absence of quantitative data on allergen type and exposure intensity, our findings lack specificity regarding particular occupational exposures. Future research should include a detailed assessment of workplace allergens.

Despite these limitations, our results have important clinical implications. Considering obesity as a potentially modifiable risk factor for OAR severity could lead to more targeted interventions. Furthermore, our study highlights the need for a multidisciplinary approach to OAR management involving collaboration between occupational physicians, allergists, nutritionists, and other healthcare professionals.

## 5. Conclusions

This study identified obesity as a modifiable factor associated with increased clinical severity of OAR. The observed association suggests the potential importance of incorporating obesity into OAR management strategies. However, given the cross-sectional design of this study, causal inferences cannot be drawn. Future longitudinal research is therefore essential to confirm these findings, elucidate the underlying biological and behavioral mechanisms, and evaluate the impact of targeted interventions aimed at reducing the obesity-related burden in populations at risk for OAR.

## Figures and Tables

**Table 1:** Sociodemographic, lifestyle, and occupational characteristics of the study population.

		Effective (n)	Percentage (%)
Gender			
	Male	68	34.7
	Female	128	65.3
Marital status			
	Married	147	75
	Single	46	23.5
	Divorced	2	1
	Widower	1	0.5
Educational qualifications			
	Illiterate	7	3.6
	Primary	136	69.4
	Secondary	38	19.4
	Superior	15	7.6
Smoking			
	Non-smoking	154	78.6
	Active	38	19.4
	Passive	4	2
Alcohol consumption			
	No	191	97.4
	Yes	5	2.6
Physical activity *			
	No	143	73
	Yes	53	27
Extraprofessional activity			
	None	180	91
	Domestic handiwork	8	4
	Gardening	6	3
	Animal husbandry	4	2
Age (Mean ± SD, extremes, years)		39.69 ± 7.92	19–59
Sector of activity			
	Textile industry	97	49.5
	Electronics Industry	29	14.8
	Agri-food sector	20	10.2
	Chemical industry	11	5.6
	Wood industry	7	3.6
	Healthcare sector	7	3.6
	Plastics industry	3	1.5
	Others	22	11.2
Profession			
	Blue-collar workers	174	88.7
	White-collar workers	16	8.2
	Others	6	3.1

* Physical activity refers to leisure-time physical activity outside of work.

**Table 2:** Medical characteristics of the study population.

			Effective (n)	Percentage (%)
**Family history of allergy**				
	No		168	85.7
	Yes		28	14.3
		Asthma	17	8.7
		Rhinitis	6	3.3
		Eczema	1	0.5
		Hives	2	1
		Allergic conjunctivitis	2	1
**Personal history of allergy**				
	No		171	87.2
	Yes		25	12.8
		Asthma	6 *	1.2
		Rhinitis	11 *	5.6
		Eczema	5 *	3.6
		Hives	4 *	3.6
		Allergic conjunctivitis	3 *	1.5
**BMI**				
	Thin		2	1
	Normal		48	24.5
	Overweight		77	39.3
	Obese		69	35.2

BMI: Body mass index, *: respondents could select more than one option.

**Table 3:** Severity of functional signs among the study population.

Functional Sign	Subjective Severity		
	Absent (%)	Light (%)	Bothersome (%)
Nasal obstruction	21.4	25	53.6
Sneezing	11.2	14.8	74
Rhinorrhea	32.2	11.2	56.6
Anosmia	92.4	5.6	2
Pruritus	14.8	21.4	63.8

**Table 4:** Clinical examination and rhinomanometry findings.

		Effective (n)	Percentage (%)
**ENT examination**			
	Normal	91	46.4
	Pathological	72	36.7
	Non-conducted	33	16.8
**Rhinoscopy**			
	Normal mucosa	11	5.6
	Pale mucosa	44	22.4
	Congestive mucosa	7	3.6
	Other aspects	9	4.6
	Non conducted	125	63.8
**Cardiopulmonary exam**			
	Normal	157	80.1
	Pathological	39	19.9
**Rhinomanometry**			
	No obstruction (>870 mL/s)	82	41.8
	Mild obstruction (700–870 mL/s)	27	13.8
	Moderate obstruction (500–700 mL/s)	34	17.3
	Severe obstruction (<500 mL/s)	53	27

ENT: Ear, nose and throat.

**Table 5:** Prevalence of different degrees of OAR in the different BMI groups.

BMI	Severity	
	PAREO > 5	Severe Obstruction (<500 mL/s)
Thin	0 (0%)	1 (1.9%)
Normal	4 (5.9%)	10 (18.8%)
Overweight	27 (39.7%)	24 (45.3%)
Obese	37 (54.4%)	18 (34%)

**Table 6:** Factors associated with the clinical severity of occupational allergic rhinitis.

		Severe OAR n (%)	Non-severe OAR n (%)	*p*	OR CI 95%
**Gender**					
	Male	33 (28.4)	35 (43.8)		
	Female	83 (71.6)	45 (56.3)	0.027	1.9 [1.07–3.5]
**Marital status (married)**		96 (82.8)	51 (63.8)	0.003	2.7 [1.4–5.2]
**Educational qualifications (Primary)**		85 (73.3)	51 (63.8)	0.15	1.5 [0.8–2.8]
**Activity sector (textile industry)**		63 (54.3)	34 (42.5)	0.10	1.6 [0.9–2.8]
**Mask wearing**		14 (12.1)	15 (18.8)	0.19	0.5 [0.2–1.3]
**Family atopy**		19 (16.4)	9 (11.3)	0.31	1.5 [0.6–3.6]
**Personal atopy**		11 (9.5)	4 (5)	0.24	1.9 [0.6–6.4]
**Smoking**		19 (16.4)	19 (23.8)	0.20	0.6 [0.3–1.2]
**Physical activity**		93 (80.2)	50 (62.5)	0.006	2.4 [1.2–4.6]
**Obesity**		57 (49.1)	12 (15)	<10^−3^	5.4 [2.6–11.1]
**Normal ENT examination**		58 (50)	33 (41.3)	0.4	**-**
		(Mean ± SD, extremes, years)			
**Age**		40.4 ± 7.7	38.6 ± 8	0.12	**-**
**Professional seniority**		16.3 ± 8.1	13.6 ± 7.8	0.024	-
**Time to onset of symptoms**		10 ± 7.4	7.7 ± 6.9	0.036	-

ENT: ear, nose and throat; OR: odds ratio; CI: confidence interval.

**Table 7:** Clinical severity of occupational allergic rhinitis and independent factors in multivariate analysis.

Variables	*p*	aOR	95% CI
Obesity	<0.001	5.47	2.6–11.1
Activity sector (textile industry)	0.001	3.8	1.8–8.3
Mask wearing	0.009	0.089	0.014–0.55

aOR: Adjusted odds ratio CI: Confidence interval.

## Data Availability

The raw data supporting the conclusions of this article will be made available by the authors upon request.
